# “My right to walk, my right to live”: pedestrian fatalities, roads and environmental features in Benin

**DOI:** 10.1186/s12889-021-10192-2

**Published:** 2021-01-19

**Authors:** Yolaine Glèlè-Ahanhanzo, Alphonse Kpozèhouen, Charles Sossa-Jerôme, Ghislain E. Sopoh, Huguette Tedji, Koovy Yete, Alain Levêque

**Affiliations:** 1 Multidisciplinary Research Unity for Road Crashes Prevention (ReMPARt), Epidemiology and Bio-statistic Department, Regional Institute of Public Health, University of Abomey-Calavi, Ouidah, Benin; 2 Health Promotion Department, Regional Institute of Public Health, University of Abomey-Calavi, Ouidah, Benin; 3 Department of Health and Environment, Regional Institute of Public Health, University of Abomey-Calavi, Ouidah, Benin; 4 National Centre for Road Safety, Cotonou, Benin; 5 Public Health School (Université Libre de Bruxelles) – Center for Research in Epidemiology, Biostatistics and Clinical Research, Brussels, Belgium

**Keywords:** Pedestrians, Mortality, Traffic crashes, Environment

## Abstract

**Background:**

The implementation of road safety interventions in many developing countries usually focuses on the behavior of users. In order to draw more attention on the role of road infrastructure and physical environment in road safety interventions, this study aims to analyze the environmental and road factors associated with the pedestrians involved in traffic crashes in Benin.

**Method:**

The method used was an analysis of national road crash statistics for the period 2008 to 2015. The information available included the circumstances surrounding the collision, the road infrastructure, the vehicles and the individuals involved. A multiple logistic regression was used to identify predictors of pedestrian mortality in traffic crashes.

**Results:**

During the period studied, 3760 crashes involved at least one pedestrian. The death rate among these pedestrians was 27.74% (CI 95%: 26.31–29.20). The mortality predictors were the area in which the crash occurred (OR = 4.94; CI 95%: 4.10–5.94), the day of the crash (OR = 2.17; CI 95%:1.34–3.52), light levels (OR = 1.30; CI 95%: 1.06–1.59), road classification (OR = 1.79; CI 95%: 1.46–2.20), the condition of the road surface (2.04, CI 95%: 1.41–2.95) and the position of the pedestrian during the crash (OR = 1.69; CI 95%: 1.19–2.38).

**Conclusions:**

These results support the need for a holistic approach to interventions aiming to tackle deaths on roads. Interventions should integrate environmental factors for greater pedestrian safety around roads with appropriate signs, roads in good condition and awareness campaigns for a proper use of road infrastructures.

## Background

In modern times, any death or injury on roads is unacceptable given that transportation is a part of economic life and all users have the right to safe travel. Road crashes are responsible for more than 1.35 million deaths per year worldwide; they are the third leading cause of death and disability in sub-Saharan Africa [1, 2]. Although walking has health benefits, the highest road traffic death rates in Africa are among pedestrians at 3.4 per 100,000 inhabitants (95% CI: 2.5–4.2), after motorized four-wheeled vehicle occupants at 5.9 per 100,000 population (95% CI: 4.4–7.4) [3]. According to the latest WHO estimates, pedestrians account for 40% of all deaths by road crashes in Africa, which are estimated at 26.6 per 100,000 inhabitants [2]. In our settings of huge urbanization with no real policy for city building, among the vulnerable road users, pedestrians are particularly exposed because of the lack of pedestrian facilities [4]. In many African countries, pedestrians account for the largest share of road traffic injuries and deaths [5–8]. The risk factors for pedestrians being hit and killed come in a variety of forms. These factors can be related to both pedestrian and antagonist behavior and are the result of deliberate violations or unintentional errors [9]. For the individual themselves, risky behaviors mentioned in the literature are linked to consumption of alcohol, chatting with other people, and use of a cell phone [10–13]. As regards infrastructure, the characteristics of the roads themselves, such as type of roadway facilities, have also been identified as risk factors involved in pedestrian deaths [14]. In urban areas, it has also been proved to some extents that the design of road infrastructure as well as the interrelationships with the environment are predictors of the occurrence of pedestrian crashes, especially at intersections [15]. Thus, the importance of the environment and infrastructure measures and the need to implement road safety measures integrating the protection of vulnerable road users such as pedestrians have also been highlighted by many other authors [8, 10, 16]. As such, the implementation of road infrastructure measures has proven to be effective in reducing deaths among pedestrians [17] and is recommended by the World Health Organization [18].

The Safe System Approach includes interventions targeting vehicles, roads, users, and speed limits [19, 20]. In Benin, where traffic rules are often not followed and where the transport system includes a large number of motorcyclists (> 80%), a large portion of road safety interventions are targeted at users, with motorcyclists in particular highlighted among vulnerable road users. These interventions, however, should not overshadow other areas of intervention, in particular road infrastructure and environment, which should be taken into consideration in a genuine road safety policy in a context where speeding is a real problem [21].

With the aim of contributing to the strategic orientations for improving the safety of vulnerable road users in Benin and pedestrians especially, this study aims to analyze the environmental and road factors associated with the pedestrians involved in traffic crashes.

## Methods

### Study area

The area of the study is the Republic of Benin. Located in West Africa, it is a coastal country covering around 115,000 km^2^ with a population of 11 million spread across 12 departments. Cotonou, the economic capital located in the Littoral department, is the center of many forms of trade, and road transport makes up the majority of transport in a country that is a strategic hub for commerce with neighboring countries in the Sahel.

### Data source

The data for this study came from the database of crashes recorded by the Benin police force for the period 2008 to 2015. This database was created at the end of the 1990s and provides information on the crash characteristics and circumstances, the state of the road infrastructure, the vehicles, and the individuals involved. The information on the individuals involved are aggregated by crash and concern those victims injured and killed (death of the victim at the crash site or during transport to a healthcare facility). Data on the age of the victims was available in the form of age bands (expressed in years) in three categories: < 13/13–20/20 and over. Information on the sex of victims was not available and the database includes no information on unharmed victims. This database contains the information gathered by the police at the crash site. Moreover, it is also managed by the national centre for road safety, called the Centre National de Sécurité Routière (CNSR), which produces the annual statistical reports [22, 23].

### Records selection and variables

The working hypothesis was that the environmental factors surrounding road infrastructure increase the probability of death immediately following the crash among pedestrians involved in a traffic collision. So, from the database, we extracted the records related to crashes with at least one pedestrian injured for the analysis, which was therefore limited to the data on crashes that resulted in at least one pedestrian harmed. We did not use any further exclusion criteria. The statistical unit of analysis was the collision; a crash with multiple pedestrian deaths and/or injuries was treated as one unique collision.

For this analysis, the safe system approach implies an integrated conceptual framework with the environment and the road, the vehicles, and the behavioral factors. We selected our variables according to their availability in the database and from reviewing the literature [10, 24, 25]. As behavioral data were not recorded, we were unable to integrate these aspects in our analysis. In the same way, there was no detail about technical issues related to the vehicle involved, so theses variables were not included. All the variables related to the environment and road infrastructure that were available were included in the analysis; we excluded the variables related to crash location recorded by GPS (Global Positioning System) coordinates, which were not comprehensive (GPS coordinates recording began in 2015). The dependent variable was the death of a pedestrian. This is a dichotomous variable, with “Yes” and “No” being the only possible responses. The death of a pedestrian was recorded as “Yes” if at least one pedestrian died following the traffic crash and “No” otherwise.

The independent variables considered related to the environmental factors surrounding the traffic collision: the area (rural/urban), the type of day (regular working day/weekend/public holiday/market day), the light levels (during day/at night with public lighting/at night without public lighting), the atmospheric (weather) conditions (normal/abnormal). For the latter, abnormal atmospheric, weather conditions were rain, fog, storm, or dust storm. Characteristics of the road infrastructure were also taken into account: road classification (national and urban roads/unclassified paths and trails/inter-country national roads known as Routes Nationales Inter-Etats (RNIEs)), traffic crash at an intersection (yes/no), road profile (flat/incline or elevation), traffic crash on a bend or curve (no/yes), type and state of road surfacing (cobblestones in good condition/asphalt in good condition/damaged condition), and presence of safety traffic barriers on the ground (yes/no). The characteristics of the collision, such as the type of vehicle involved (motorized with two wheels/light four-wheeled vehicle/truck) and the position of the pedestrian during the crash (crossing an intersection/crossing away from an intersection/on the road or surrounding area/pedestrian outside of the primary collision) were included. For this last variable, pedestrians outside of the primary crash were those who were injured as a result of the damage caused by the collision.

### Statistical analysis

The data were processed using Stata 15. The standard descriptive statistics were presented with a comparison between the qualitative independent variables and the dependent variable using the Pearson chi-square test. The factors linked to the death of pedestrians were selected at a threshold of 20% using the single-variate analysis and were added to a multivariate logistic regression model using a descending stepwise procedure. The associations were assessed using the odds ratio (OR) with a confidence interval of 95% (CI 95%). The suitability of the final model was tested using the Hosmer and Lemeshow test. The 5% threshold was selected for determining statistical significance.

## Results

### Descriptive characteristics of the crashes and pedestrians involved

Out of the 42,846 crashes recorded during the period, 3760 involved at least one pedestrian, amounting to 8.78%. A total of 4392 pedestrians were involved, with 3284 wounded and 1108 killed. The age group of subjects 20 years and older (*n* = 2.396) represents 54.55% of victims and those aged under 13 years represents 26.23% (*n* = 1.152).

Close to half (43.67%) of crashes were recorded in the Littoral department. The other departments each accounted for less than 10%. Most of the crashes occurred in an urban area (70.19%). In the same way, a significant proportion occurred on a regular working day (69%). Normal atmospheric conditions were the most reported (95.95%). In 50.51% of cases, the road infrastructure involved was an RNIE. Crashes away from bends/curves were the most frequent (91.17%). The pedestrian was crossing away from an intersection in 11.55% of cases or walking on the road or surrounding area in 48.8% of cases. The road had an asphalt surface in good condition in 67.71% of cases, and safety traffic barriers were present in 33.08% of cases. In 57.78% of cases, the crash involved a light four-wheel vehicle (Table 1).

**Table 1:** Characteristics of the crashes involving pedestrians in Benin, 2008–2015

Variables	Number (n)	Percentage (%)
**Environmental factors**		
***Area***	3760	
Urban	2639	70.19
Rural	1121	42.48
***Type of day***	3681	
Regular working day	2576	69.98
Weekend	865	23.50
Public holiday	96	3.73
Market day	144	3.91
***Atmospheric conditions***	3751	
Normal	3599	95.95
Abnormal	152	4.05
***Light levels***	3760	
During day	2674	71.12
At night with public lighting	282	7.50
At night without public lighting	804	21.38
**Characteristics of the road infrastructure**		
***Road classification***	3760	
National and urban roads	1588	42.23
Unclassified paths and trails	273	7.26
Inter-country national roads – RNIE	1899	50.51
***Traffic crashes at an intersection***	3757	
Yes	592	15.76
No	3165	84.24
***Road profil***	3757	
Flat	3580	95.29
Incline or elevation	177	4.71
***Traffic crashes on a bend or curve***	3760	
No	3428	91.17
Yes	332	8.83
***Type and state of road surfacing***	3757	
Cobblestones in good condition	604	16.08
Asphalt in good condition	2544	67.71
Damaged condition	609	16.21
***Presence of safety traffic barriers on the ground***	3742	
Yes	1238	33.08
No	2504	66.92
**Characteristics of the collision**		
***Type of vehicle involved***	3733	
Motorized with two wheels	1145	30.67
Light four-wheeled vehicle	2157	57.78
Truck	431	11.55
***Position of the pedestrian during the collision***	3437	
Crossing an intersection	645	18.77
Crossing away from an intersection	397	11.55
Road and surrounding areas	1680	48.88
Pedestrian outside of the primary collision	715	20.80

### Prevalence of crashes with death of pedestrians and associated factors

Out of the 3760 crashes involving pedestrians, the number of crashes with at least one pedestrian fatality was 1,043; thus the death rate of pedestrians was 27.74% (CI 95%: 26.31–29.20).

The results show a significantly higher proportion of pedestrian fatalities in crashes in rural areas compared to those in urban areas. In addition, crashes occurring on public holidays or market days were related to a significantly higher proportion of pedestrian fatalities compared to crashes occurring on regular working days. With respect to brightness, there was a significant difference in the proportion of pedestrian fatalities observed in crashes occurring at night without street lighting compared to those occurring during the day. Similarly, during crashes under abnormal weather conditions, the results show a significantly higher proportion of pedestrian fatalities compared to crashes under normal weather conditions. In addition, crashes on RNIEs resulted in higher proportions of pedestrian fatalities compared to national and urban roads, as well as crashes on damaged roads compared to cobblestones in good condition. We also observed a higher proportion of pedestrian fatalities in crashes that occurred during an attempt to cross outside an intersection compared to crashes that occurred during intersection crossing (Table 2).

**Table 2:** Associated factors of death among pedestrians involved in a traffic crashes in Benin. 2008–2015: Univariate and multivariate analysis

Variables	Number of crashes (N)	Number of crashes with death of a pedestrian (n)	Percentage (%)	OR brut	95%CI	***p***-value	AOR	95%CI	***p***-value
**Environmental factors**									
***Area***	3760					0.000			0.000
Urban	2639	441	16.71	1.00			1.00		
Rural	1121	602	**53.70**	5.78	4.95–6.76		4.94	4.10–5.94	
***Type of day***	3681					0.005			
Regular working day	2576	665	25.82	1.00			1.00		
Weekend	865	250	28.90	1.17	0.98–1.39		1.17	0.96–1.43	0.119
Public holiday	96	37	38.54	1.80	1.18–2.74		2.17	1.34–3.52	0.020
Market day	144	58	40.28	1.94	1.37–2.74		1.55	1.04–2.31	0.031
***Atmospheric conditions***	3751					0.002			
Normal	3599	982	27.29	1.00					
Abnormal	152	54	**35.53**	1.47	1.05–2.06				
***Light levels***	3760					0.013			
During day	2674	715	26.74	1.00			1.00		
At night with public lighting	282	72	25.53	0.98	0.71–1.24		1.32	0.94–1.85	0.109
At night without public lighting	804	256	**31.84**	1.28	1.08–1.52		1.30	1.06–1.59	0.012
**Characteristics of the road infrastructure**									
***Road classification***	3760					0.000			
National and urban roads	1588	267	16.81	1.00			1.00		
Unclassified paths and trails	273	90	32.97	2.43	1.83–3.23		1.25	0.85–1.82	0.260
Inter-country national roads - RNIE	1899	686	**36.12**	2.80	2.38–3.29		1.79	1.46–2.20	0.000
***Traffic crashes at an intersection***	3757					0.000			
Yes	592	105	17.74		1.00				
No	3165	935	**29.54**	1.95	1.55–2.43				
***Road profil***	3757					0.000			
Flat	3580	970	27.09		1.00				
Incline or elevation	177	71	**40.11**	1.80	1.32–2.46				
***Traffic crashes on a bend or curve***	3760					0.012			
No	3428	931	27.16						
Yes	332	112	**33.73**	1.37	1.07–1.74				
***Type and state of road surfacing***	3757					0.000			
Cobblestones in good condition	604	68	11.26	1.00			1.00		
Asphalt in good condition	2544	757	29.76	3.34	2.56–4.36		1.29	0.93–1.79	0.134
Damaged condition	609	216	**35.47**	4.33	3.20–5.86		2.04	1.41–2.95	0.000
***Presence of safety traffic barriers on the ground***	3742					0.000			0.019
Yes	1238	419	33.84	1.00			1.00		
No	2504	617	24.64	0.64	0.55–0.74		0.80	0.66–0.96	
**Characteristics of the collision**									
***Type of vehicle involved***	3733					0.000			
Motorized with two wheels	1145	234	20.44	1.00					
Light four-wheeled vehicle	2157	608	28.19	1.53	1.29–1.81				
Truck	431	190	**44.08**	3.07	2.42–3.90				
***Position of the pedestrian during the collision***	3437					0.000			
Crossing an intersection	645	132	20.47	1.00					
Crossing away from an intersection	397	79	19.90	0.96	0.71–1.32		1.00		
Road and surrounding areas	1680	474	28.21	1.53	1.23–1.90		1.69	1.19–2.38	0.003
Pedestrian outside of the primary collision	715	242	33.85	1.99	1.55–2.54		1.91	1.49–2.44	0.000

*p value HL: 0.4171

### Predictors of death among pedestrians involved in a traffic crash

The multivariate analysis showed that the area where the traffic crash occurred, the day, light levels, road classification, state of the road surface and the position of the pedestrian during the traffic crash were predictors for the death of a pedestrian.

As such, after adjusting for the other variables, the probability of death of a pedestrian in a crash was five times higher in rural areas than in urban areas (OR = 4.94; CI 95%: 4.10–5.94). The probability of death of a pedestrian was higher in crashes occurring on public holidays (OR 2.17; CI 95%: 1.34–3.52) and market days (OR = 1.55; CI 95%: 1.04–2.31) compared with those occurring on regular working days. Crashes occurring at night without public lighting had a higher probability of death of a pedestrian than those occurring during the day (OR = 1.30: CI 95%: 1.06–1.59). Crashes occurring on an RNIE had twice the probability of death of a pedestrian compared to those on national and urban roads with an OR of 1.79 (CI 95%: 1.46–2.20). Similarly, those occurring on a damaged road had twice the probability of death of a pedestrian compared to those on cobblestones in good condition with an OR of 2.04 (CI 95%: 1.41–2.95). All other things being equal, the probability of death of a pedestrian was higher in crashes where the pedestrian was attempting to cross away from an intersection and when the pedestrian was on the road or surrounding area with an OR of 1.69 (CI 95%: 1.19–2.38) and 1.91 (CI 95%: 1.49–2.44) respectively compared with crashes in which pedestrians were not directly involved in the principal crash (Table 2).

## Discussion

The aim of this study was to identify environmental and infrastructure predictors for deaths among pedestrians involved in a road crash in Benin, in a context where a holistic view is now recommended in the management approach to road safety [2, 26]. The results have shown that environmental factors such as the area and day on which the traffic crash occur, the light levels and the characteristics of the infrastructure, such as the classification of the road, state of the road surface and position of the pedestrian during the traffic crash, are predictors for the death of a pedestrian.

The number of crashes involving pedestrians and the proportion of deaths are higher than in a similar study conducted in Ghana in 2019 on crash data covering the period 2007 to 2016 [13]. That study looked at 328 traffic crashes between 2007 and 2016, of which 51 involved the death of a pedestrian, making up 15.51%. These statistics are lower than those reported in this study. The studies, however, are not comparable due to the differences in the territories examined and the economic context: surrounding transport specifically. Nevertheless, there is agreement on the fact that the reliability of the figures depends on the capacity of the collection system to capture the events, in particular the crash and the deaths. In most cases, low-income countries still have crash data collection systems that perform poorly, in particular as regards the completeness of the data. There are many reasons for this, such as limited integration with hospital systems [27–29]. The low proportion of deaths may also be partially explained by how the immediate post-crash healthcare system operates. Studies have shown that pre-hospital mortality from traffic crashes is lower according to the time taken to receive care and the quality of the pre-hospital system [30–32]. In our contexts, the availability and correct functioning of these services is not guaranteed; however, they are essential for reducing morbidity and mortality linked to traffic crashes. An assessment conducted in Nigeria showed inadequate quality in the implementation of immediate post-crash services with delays in receiving treatment and inadequate pre-hospital care, with fewer than 30% of victims of traffic crashes receiving adequate pre-hospital treatment [33].

Although the proportions observed differ, the results of this study agree with the literature on the fact that the majority of crashes involving pedestrians occur in urban areas and do not occur at intersections. According to a study in Ghana for the period 2008 to 2015, over 72% of crashes involving pedestrians occurred away from an intersection [13]. According to another study conducted in Israel, the majority of crashes (95%) and pedestrian deaths (75%) occurred in urban areas and the majority of deaths from traffic crashes occurred away from intersections [34]. In this same study, the majority of crashes (77%) and pedestrian deaths (81%) occurred when crossing the road. These figures are close to those obtained in this study and those reported in Ghana, with 70% of traffic crash-related pedestrian deaths occurring when crossing the road [5]. Thus, crossing the road is an act that exposes pedestrians to significant risk. The fact that the majority of crashes and deaths occur away from intersections may be explained here by the fact that pedestrians look to cross away from intersections out of caution, given the lighter flow of traffic and fewer interactions. Moreover, in a context where, according to national statistical data, less than 5% of roads are paved nationwide [23], there are very few pedestrian crossings in the road infrastructure in Benin, which is not the case in Israel, where most pedestrian crossings are marked and signposted [34]. Despite this, 22% of pedestrian deaths occur on pedestrian crossings, which highlights, among other factors, the issues of speeding and driver awareness. These observations raise important questions and assert the need, through a global approach, for strategies adapted to the characteristics of the road infrastructure (type, quantity and position of signage, number of intersections, design of pedestrian crossings) related to the safety of pedestrians and raising awareness among all users (pedestrians and drivers) on road use and behaviors to adopt. Thus, for example, certain authors have shown that areas with many intersections have a lower risk of crashes with pedestrians [35].

Although the crashes were predominantly in urban areas, our results show the probability of death to be five times higher in rural areas than in urban areas. These results are corroborated by the literature and may be explained by less access to healthcare services and thus a delay in receiving treatment following a crash [6, 36]. In fact, although there are emergency phone numbers to dial in order to alert the fire department or the police in case of road crash, there is no pre-hospital care structure service for road crashes victims with national coverage. Furthermore, as in most developing countries, accessibility to health care is characterized by a disparity between rural and urban areas [37]. A human factor is also mentioned by certain authors to explain the higher risk of post-crash death in rural areas, linked to lower risk perception by the pedestrians [38]. Regardless of whether it is in urban or rural areas, the behaviors of users and especially pedestrians, with respect to their risk perception, influence the risk of traffic crashes and deaths [39, 40]. Speeding could also go toward explaining this difference, but speed data were not recorded and therefore were not available in the database. This is a limitation of the analysis and shows the challenge of working with administrative data that most often do not integrate all information needed for a thorough analysis. All these factors should be incorporated into a comprehensive vision for developing other interventions, including those relating to road infrastructure. This also confirms the need to consider interventions aimed at improving individuals’ perception of traffic risks, user vulnerability, and practices and behaviors on the road to improve safety for everyone.

The literature also agrees on the role of light levels in crashes for all users, especially the risks incurred by pedestrians [7, 36, 41, 42]. The results of this study suggest the same, with the observed probability of death being higher for pedestrians in traffic crashes occurring at night without public lighting. In Benin, where public lighting has the dual issues of limited existence of lighting on major highways and extremely low actual operating ratio of this lighting where it does exist, it is essential that the public authorities take note of the protective effect of public lighting and incorporate it into interventions to improve user safety on roads.

With respect to specific days, there is no real consensus on the role of the weekend as a risk factor for traffic crashes [6, 7]. The results of this study instead point to a higher probability of mortality for pedestrians on public holidays and market days. This may be explained by excesses linked to public holidays and that lead to various risk behaviors (speeding, loss of control, improper crossing of roads). Markets are sporadic areas with high population density which, in the socio-cultural context of Benin, are mainly held alongside roads, despite measures to raise awareness among vendors who leave their reserved location within the designated market area. This situation exposes pedestrians (sellers and buyers) to risk due to the high population density [35] and especially being alongside major highways such as RNIEs. Major highways have been identified as risk factors for traffic crashes and deaths for pedestrians [4, 41]. Our results point in the same direction and may be explained by the potential for speeding on these wide roads in a context where there is limited enforcement, allowing users (over 70%) to travel frequently and significantly above the speed limit [21]. Beyond the type of road, the characteristics of the road surface were also mentioned as a factor related with the probability of death of pedestrians [14, 43]. This is confirmed from these results which show that a damaged surface doubles the risk of pedestrian death compared with a surface in good condition. It is also obvious that in a context of very poor quality of road infrastructure like the case of Benin [23], there is a need of greater consideration by public authorities of the role of road infrastructure quality on user protection and safety.

### Limitations

This study reports results that are of interest for decision-making given that they apply to the entire country, reflect circumstances over multiple years, is the first analysis of this type and, above all, examines a group that is known to be vulnerable but which is little studied within the country. The limitations of the study, however, are the fact that it had to be restricted to variables available in the database. As such, with the age (individual data) and sex of the pedestrians not available, an analysis could not be performed of the groups at risk. Other factors such as road characteristics (pedestrian crossing markings and signage), vehicle characteristics, speed data and other behavioral data could also be worth including in the analysis. Furthermore, underreporting and selection bias cannot be excluded because of possible disparities between data collection (urban vs. rural areas or casualties vs. no casualties), or non-integration of hospital data. Most of the time, road crashes data are limited by lack of comprehensiveness and are not reliable enough to conduct analyses that integrate all the components involved in the occurrence of road crashes. Furthermore, it is tough to integrate some important methodological issues as crash predictors reliability or responsibility, culpability in crash occurrence [44]. To improve this situation, suggestions could be made to the CNSR to update their database to improve the availability of relevant data for analyzes to assist with decision-making. Moreover, an integrative approach with national health information systems is needed for a better analysis.

## Conclusion

This study has shown that the characteristics of the environment and the infrastructure, such as road classification, state of road surface and position of the pedestrian during traffic crashes are predictors for the death of pedestrians involved in traffic crashes in Benin. These factors should be considered when developing planning policies and effective interventions to increase safety for pedestrians in Benin. Thus, in practical terms, particular emphasis should be placed on guaranteeing greater pedestrian safety around RNIE, ensuring that the road surface is in good condition and that signs are adapted to facilitate pedestrian crossings; all interventions should be accompanied by awareness campaigns for all road users.

## Data Availability

The dataset used and analyzed during the current study are available from the National Centre of Road Safety (CNSR).

## Abbreviations

CNSR: National Centre for Road Safety
GPS: Global Positioning System
RNIE: inter-country national roads
